# Delayed Activation Kinetics of Th2 and Th17 Cells Compared to Th1 Cells

**DOI:** 10.3390/cells6030029

**Published:** 2017-09-12

**Authors:** Andrea Duechting, Anna Przybyla, Stefanie Kuerten, Paul V. Lehmann

**Affiliations:** 1 R & D Department CTL, Shaker Hts, OH 44122, USA; andrea.duchting@immunospot.com (A.D.); anna.przybyla@immunospot.com (A.P.); 2 Department Anatomy, University Erlangen, 91054 Erlangen, Germany; stefanie.kuerten@fau.de

**Keywords:** cytokine kinetics, ELISPOT, CD4 cells, T cells, immune monitoring, multiplexing

## Abstract

During immune responses, different classes of T cells arise: Th1, Th2, and Th17. Mobilizing the right class plays a critical role in successful host defense and therefore defining the ratios of Th1/Th2/Th17 cells within the antigen-specific T cell repertoire is critical for immune monitoring purposes. Antigen-specific Th1, Th2, and Th17 cells can be detected by challenging peripheral blood mononuclear cells (PBMC) with antigen, and establishing the numbers of T cells producing the respective lead cytokine, IFN-γ and IL-2 for Th1 cells, IL-4 and IL-5 for Th2, and IL-17 for Th-17 cells, respectively. Traditionally, these cytokines are measured within 6 h in flow cytometry. We show here that 6 h of stimulation is sufficient to detect peptide-induced production of IFN-γ, but 24 h are required to reveal the full frequency of protein antigen-specific Th1 cells. Also the detection of IL-2 producing Th1 cells requires 24 h stimulation cultures. Measurements of IL-4 producing Th2 cells requires 48-h cultures and 96 h are required for frequency measurements of IL-5 and IL-17 secreting T cells. Therefore, accounting for the differential secretion kinetics of these cytokines is critical for the accurate determination of the frequencies and ratios of antigen-specific Th1, Th2, and Th17 cells.

## 1. Introduction

The immune system provides protection from various infections and tumors, but can also mediate allergies, autoimmunity and transplant rejection. Over the last decades, it has become evident that in each of these cases different types of effector T cell classes play a role, the primary ones being Th1, Th2, and Th17 [1]. Th1 cells mediate their effector functions via the secretion of interferon gamma (IFN-γ) that inhibits viral replication in infected cells, and upregulates major histocompability complex (MHC) antigen expression on such cells priming them for T cell recognition. Th2 cells secrete IL-4 that is involved in regulating antibody-mediated immunity by controlling immune globulin class-switching. A sublineage of Th2 cells is involved in anti-parasite defense via the secretion of IL-5. Finally, the Th17 cell type mediates delayed type hypersensitivity (DTH) by secreting IL-17 [2]. In DTH, macrophages are recruited and activated to constitute the primary line of defense against intracellular pathogens. 

The Th1, Th2 and Th17 lineages emerge through instructed differentiation. Naïve T cells are uncommitted and can develop into either of these cell types [3]. During the primary immune response, when naïve T cells first encounter antigen, they start to proliferate, and dependent on the cytokine environment in which this reaction occurs they also engage in differentiation. In the presence of IL-12, Th1 cells emerge while in the presence of IL-4 Th2 cell development occurs [4,5,6]. If IL-6 and TGFβ prevail in the microenvironment, Th17 cells emerge [7]. Once Th1/Th2/Th17 differentiation is completed, which happens within the first 10 days of the primary immune response [8], the cytokine expression profiles of these cells are firmly imprinted and mutually exclusive [9]. Upon antigen re-encounter, Th1 cells will secrete IFN-γ (but no IL-4 or IL-17), Th2 cells will produce IL-4 or IL-5, but no IFN-γ or IL-17, and Th17 cells IL-17 in the absence of these other cytokines [10,11]. In this way, dependent on the ratios of antigen-specific Th1/Th2 and Th17 generated, the T cell system can elicit highly specific effector mechanisms on the site of antigen re-encounter. 

Beyond mounting a T cell response per se, the magnitude of clonal expansion within the Th1/Th2/Th17 lineages is a primary factor defining whether protective immunity develops. Engaging, for example, Th2 immunity when Th1 or Th17 would be the adequate class of response can be deleterious for the host [12]. Also, allergies and autoimmunity can be viewed primarily as the consequence of engaging an inappropriate T cell effector class [13]. Therefore, our abilities to unambiguously define frequencies of Th1, Th2 and Th17 cells is at the heart of any immune monitoring effort aiming at better understanding immune-mediated processes, whether beneficial to the host, or harmful. The inclusion of cytokines other than IFN-γ into immune monitoring efforts can also be critical for identifying disease activity states. For example, it has been shown that IFN-γ ELISPOT (enzyme-linked immunospot) assays alone are not suited to distinguish between the active and latent form of tuberculosis infection in children, however, additional IL-2 testing helps to make this discrimination [14].

Detecting antigen-specific Th1/Th2 and Th17 cells relies on measuring the lead cytokines these cells produce following antigen exposure. Resting Th1, Th2 or Th17 cells, like memory cells in general, do not constitutively secrete cytokine but are preoccupied with recirculation in the body while seeking antigen [15]. Such cells are the typical lymphocytes seen in peripheral blood with a prevalent nucleus and underdeveloped cytoplasm and endoplasmic reticulum (ER) enabling protein synthesis. Upon re-encounter with antigen, these memory T cells undergo blast transformation during which their ER is re-developed, and they gain the ability to express quantities of cytokine. For Th1 cells, it is well established that it takes 12–24 h after antigen re-encounter before IFN-γ production reaches its peak [16]. In mice induction of IL-4, IL-5 and IL-17 by the corresponding T cell subset also peaks within 24 h [17]. In humans, however, there have been occasional observations of delayed IL-4, IL-5 and IL-17 secretion kinetics by memory T cells [18], but the detection of these effector T cell classes in PBMC has mostly been unsuccessful, likely because of the low frequency of such T cells, and possibly because too short antigen-stimulation cultures have been used. A systematic study to this extent is missing so far, and is presented in this report. The differences between mouse and humans might be species dependent, but might also reflect on the time elapsed since the last antigen encounter of the memory cells [19]. In mice, recall responses are studied within weeks, maximally months after the immunization; in humans, years or decades might have elapsed between the last antigen encounter of the T cell and its reactivation for the testing purpose. 

Cytokine production of antigen-specific Th1/Th2/Th17 memory cells is best studied in freshly isolated PBMC and relies on techniques permitting analysis at the single cell level. Intracytoplasmic cytokine staining (ICS) in conjunction with flow cytometry is one frequently used approach to accomplish this goal [20]. ICS has a detection limit of around 1 in 1000 events, that is 0.1% [20]. Antigen-specific Th2 and Th17 cells, however, rarely reach frequencies of even 0.1% in PBMC, and therefore mostly go undetected by ICS. Better suited for the Th1/Th2/Th17 delineation of memory cells is ELISPOT [21]. In this approach, PBMC are plated on a polyvinylidene fluoride (PVDF) membrane that has been pre-coated with cytokine-specific capture antibody. When the PBMC are present between 100,000 and one million cells per well, they form a monolayer on the membrane and contacts between T cells and antigen presenting cells (APC) are secured [22]. Once antigen is added, the antigen-specific T cells become activated and start to secrete the cytokine that they were preprogrammed to express while the cytokine is captured around the secreting cell by the cytokine-specific capture antibody on the membrane. Thus, each cytokine secreting cell leaves behind on the membrane a “cytokine spot” that can be visualized via addition of a cytokine-specific detection antibody. Counting these “spots”, also called spot-forming units (SFU), permits detection of the individual antigen-specific T cells secreting a particular cytokine, and the SFU count per well establishes the frequency at which those antigen-specific T cells occur within all PBMC plated in that well. As every cytokine producing cell is visualized, with 400,000 PBMC plated per well (as was done in this particular study), the detection limit of the ELISPOT assay is 1 in 400,000 cells, that is, the ELISPOT assay as performed was 400 times more sensitive than ICS. For ICS, toxic secretion inhibitors need to be added to retain the cytokine in the cell, limiting the antigen stimulation period to 6–8 h. In ELISPOT assays, no such additional reagents are used, and subsequently the antigen challenge period can be readily expanded to several days. Relying on the sensitivity of the ELISPOT assay and extending the observation period after antigen challenge we set out to optimize the detection of rare antigen-specific Th2 and Th17 cells. 

In the present study, we selected 12 common recall antigens. Five of them were proteins that need to be processed by APC before they are presented on MHC class II molecules to CD4 cells [23]. These are dust mite antigen (DM), purified protein derivate of mycobacterium tuberculosis (PPD), ultraviolet light (UV)-inactivated, so called “grade 2” HCMV virions (CMV gr.2), gamma radiation inactivated mumps virions, and mosquito antigen. In addition, pools of 15 amino acid long peptides were used that cover the protein sequence in 11 amino acid overlaps. Such peptide pools were used for the following proteins: BZLF1 and EBNA1 (both open reading frames of the Epstein Barr Virus, EBV), MP1H3N2, H1N1 and NPH3N2 (all three are proteins of flu virus). Finally, we also tested a 15-mer peptide pool that covers the CMV pp65 antigen sequence. Due to their length, 15-mer peptides activate CD4 cells [24]. After screening a library of healthy human donors to identify individuals who possess Th1/Th2 and Th17 memory cells specific for these antigens, we set out to study the kinetics of antigen-triggered IFN-γ, IL-2, IL-4, IL-5 and IL-17 production by the respective CD4 memory cell subset. The question was asked whether kinetics differ between individual donors and antigens. The data showed that, invariably, IL-4, IL-5 and IL-17 secretion by CD4 cells is delayed by several days compared to the production of IFN-γ. This notion has profound implications for reliable detection of Th2 and Th17 cells as these T cell subsets would go undetected in assays that do not extend an 8 h antigen stimulation period. Accounting for the delayed secretion kinetics of Th2 and Th17 cells, therefore, should be part of any immune monitoring approach. 

## 2. Materials and Methods

### 2.1. PBMC Donors

PBMC from healthy human donors were selected from the ePBMC library (CTL, Shaker Heights, OH, USA). PBMC were thawed following an optimized protocol for the recovery of viable and functional cells [25]. The cells were tested within 2 h of thawing because we found that “overnight resting” does not improve the functionality of properly cryopreserved PBMC [26] and it was reported that overnight resting changes the cytokine signatures of antigen-specific T cells [27]. Viability of the thawed cells exceeded 95% for all PBMC samples. The PBMC were resuspended at a final concentration of 4 × 10^6^ PBMC/ml in CTL-Test Medium (CTLT-005, from CTL) of which 100 μL (400,000 cells) were plated per well into the ELISPOT assay.

### 2.2. Antigens

Sixteen antigens were used in total for this study, namely: CMVA (pp65) consisting of a pool of 138 peptides derived from a peptide scan (15-mers with 11 aa overlap) through the 65 kDA phosphoprotein (pp65) of human cytomegalovirus, CMV. It was purchased from JPT (Berlin, Germany, cat. #: PM-PP65-1) and was used at the final concentration of 0.25 μg/mL. CMV pp65(495–503) is an HLA-A2-restricted immune dominant peptide of CMV [28]. It was from CTL (cat. #: CEF32-07-005), and was used at a final concentration of 1 μg/mL. CMV gr. 2 antigen: UV inactivated CMV purchased from Microbix (Mississauga, Ontario, Canada, cat. #: EL-01-02-001) and tested at a final concentration of 30 μg/mL. P3H3 antigen (from Microbix, cat. #: EL-16-03-001) representing inactivated Epstein–Barr Virus, EBV. It was tested at 30 μg/mL. Two 15-mer peptide pools of the EBV system were also used, both from JPT and at a concentration of 0.25 μg/mL: EBNA1 (PM-EBV-EBNA1, 158 peptides) and BZLF1 (cat. #: PM-EBV-BZLF1, 59 peptides). Influenza A gr. 2 antigen (from Microbix, cat. #: EL-13-02-001, used at 12.5 μg/mL). Pep Mix Influenza A NP(H3N2) is a pool of 122 15-mer peptides derived from a peptide scan through the nucleocapsid protein of Influenza A virus (from JPT, cat. #: PM-INFA_NP, used at a final concentration of 1 μg/mL). Pep Mix Influenza A MP1 (H3N2) antigen is a pool of sixty-one 15-mer peptides that cover the amino acid sequence of Influenza A virus matrix protein 1 (from JPT, cat. #: PM-INFA_MP1, final concentration 1 μg/mL). Pep Mix Influenza A HA/California (H1N1) antigen, a pool of 139 15-mer peptides deduced from a peptide scan through the hem agglutinin molecule of Influenza A virus (JPT, cat. #: PM-INFA-HACal final concentration 1 μg/mL). Mumps gr.2 antigen (from Microbix, cat. #: EL-06-02-001) was tested at a final concentration of 10 μg/mL. Dust mite antigen Mite Mix (from GreerLabs, Lenoir, NC, cat. #: B03) was tested at a final concentration of 10 μg/mL. Mosquito antigen (from GreerLabs, cat. #: B10) was tested at 5 μg/mL. PPD (from Staten Serum Institute, Copenhagen, Denmark was tested at a final concentration of 20 μg/mL. CPI (from CTL, cat. #: CTL-CPI-001) is a CD4 cell-positive control that elicits recall responses in all healthy donors. It is a pool of protein antigens derived from CMV, influenza and parainfluenza viruses, and was used at the final concentration of 6.25 μg/mL. CEF pp+ consists of a pool of 32 immune dominant nonamer peptides of CMV, EBV and flu viruses [29]—this peptide pool was from CTL (cat. #: CTL-CEF-002) and was used at a final concentration of 0.25 μg/mL.

### 2.3. Human Cytokine ELISPOT Assays

All cytokine ELISPOT assays were done using single-color enzymatic ImmunoSpot^®^ kits from CTL for detection of human IFN-γ (cat. #: CTL-HIFNG-1/5M), IL-2 (cat. #: CTL-HIL2-1/5), IL-4 (cat. #: HIL4-1/5), IL-5 (cat. #: HIL5-1/5) and IL-17 (cat.#: CTL-HIL17-1/5). Test procedures followed the manufacturer’s recommendations. In brief, antigens were plated in triplicate wells to the capture antibody precoated assay plate at a final volume of 100 μL per well with the antigen concentrations specified above for each. All antigens were dissolved in CTL Test Medium (CTLT-005). This medium alone constituted the negative control wells. The plates with the antigen were stored at 37 °C in a CO_2_ incubator until the cells were ready for plating. PBMC were added at 400,000 cells/well in 100 μL using wide-bore pipette tips. Plates were gently tapped on each side ensuring even distribution of the PBMC as they settle. The PBMC were cultured with the antigens for the time periods specified in the figures while keeping them at 37 °C and 9% CO_2_ in an incubator. After removing the cells, addition of detection antibody, and enzymatic visualization of the plate-bound cytokine, the plates were air-dried prior to analysis.

ELISPOT plates were analyzed using an ImmunoSpot S6 Ultimate Reader by CTL. Spot Forming Units (SFU) were automatically calculated by the ImmunoSpot^®^ Software for each antigen stimulation condition and the negative control wells using the Autogate^TM^ function to set up objective lower and upper gates for each cytokine [30]. 

### 2.4. Statistical Analysis

ELISPOT counts follow Gaussian (normal) distribution among replicate wells which permits the utilization of parametric statistics, including the Student’s *t*-test for identifying positive responses [31]. Accordingly, the Student’s *t*-test was done comparing SFU in the three antigen-containing replicate wells, vs. the spot counts in the three medium control wells. A *p* value < 0.05 was considered as the cut off for positivity. 

## 3. Results

### 3.1. Identifying Donors Exhibiting IFN-γ, IL-2, IL-4, IL-5 and IL-17 Recall Responses to Select Antigens

**Figure 1:**
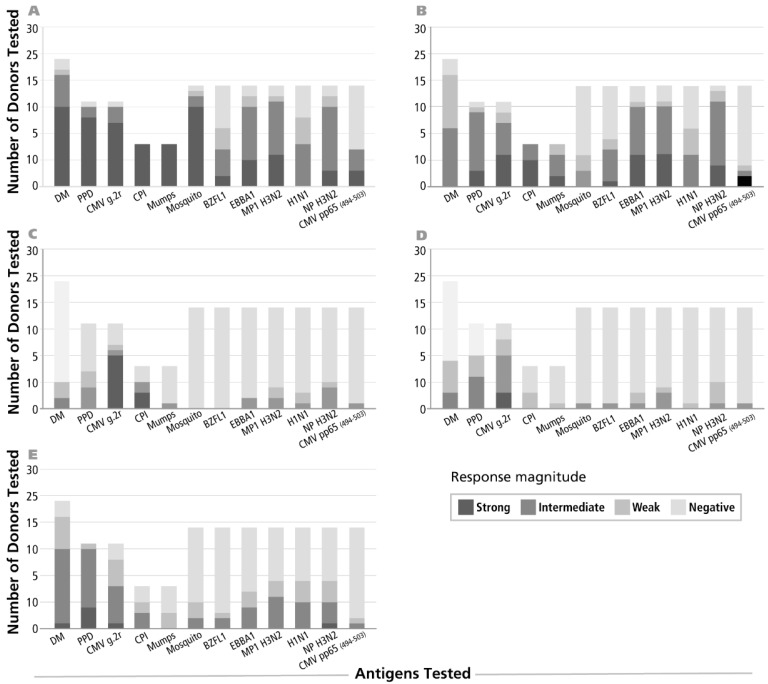
Gradation of magnitudes for antigen-specific recall responses. Peripheral blood mononuclear cells (PBMC) of healthy human donors were tested for recall responses to the antigens specified on the *X* axis—the numbers of PBMC donors tested is specified on the *Y* axis. In panel (**A**) the results for IFN-γ are shown; in (**B**–**E**) for IL-2, IL-4, IL-5 and IL-17, respectively. Response magnitudes are indicated by different shades, as specified, and are defined as follows: negative, off-white: no statistically significant difference between three medium control wells and the three antigen wells tested, as defined by the Student’s *t*-test, and a cut-off value of *p* > 0.05. Weak response, in light grey: spot counts reaching statistical difference, but less than 20 SFU per 400/000 PBMC. Intermediate response, in dark grey: Spot Forming Unit (SFU) counts for antigen-induced response between 20 and 100. Strong response, in black: more than 100 antigen-induced SFU/400,000 cells.

IFN-γ recall responses have been commonly observed. In contrast, only occasional donors have been reported to generate IL-4, IL-5 and IL-17 recall responses to select antigens and they showed a delayed kinetic [18]. We therefore set out to identify antigen/donor combinations suitable for the detection of Th2 and Th17 memory cells. PBMC of random healthy donors were tested for the twelve recall antigens specified in Figure 1. Single color ELISPOT assays detecting cells secreting IFN-γ, IL-2, IL-4, IL-5 and IL-17 were performed with 24, 48 and 72 h antigen stimulation cultures. Figure 1 summarizes the results for the respective peak of cytokine production detected (24 h for IFN-γ and IL-2, 48 h for IL-4, and 72 h for IL-5 and IL-17). A response was judged positive if in the Student’s *t*-test the comparison of the three antigen-triggered replicate wells with the three negative control wells reached a significance level of *p* > 0.05. Response magnitudes were graded as specified in the figure. In general, Th2 and Th17 cells specific for any of the antigens tested occurred in substantially lower frequencies than Th1 cells specific for the same antigen. For example, 95% of the donors displayed mosquito specific IFN-γ memory cells, while only 26% of these donors also exhibited a Th17 component, and the latter occurred in much lower SFU frequency in positive donors (see below). 

The number of PBMC donors that responded with IL-4 or IL-5 production to the individual antigens was even lower than for IL-17. For example, less than 25% of the donors showed an IL-4 recall response to antigens dust mite, mumps, EBNA1, BZLF1, MP1 H3N2, H1N1, CMV pp65 and mosquito antigens (Figure 1). While most donors exhibited IFN-γ producing memory cells in the high and intermediate frequency range (exceeding 50 SFU/400,000 PBMC) the numbers of T cells producing IL-4, IL-5 and IL-17 was prevalently in the low frequency range (less than 50 SFU/400,000 PBMC). 

### 3.2. Kinetics of the IL-17 Recall Response

**Figure 2:**
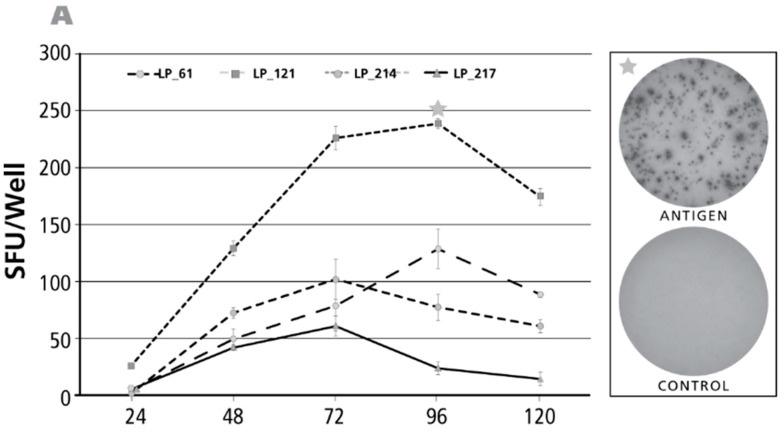

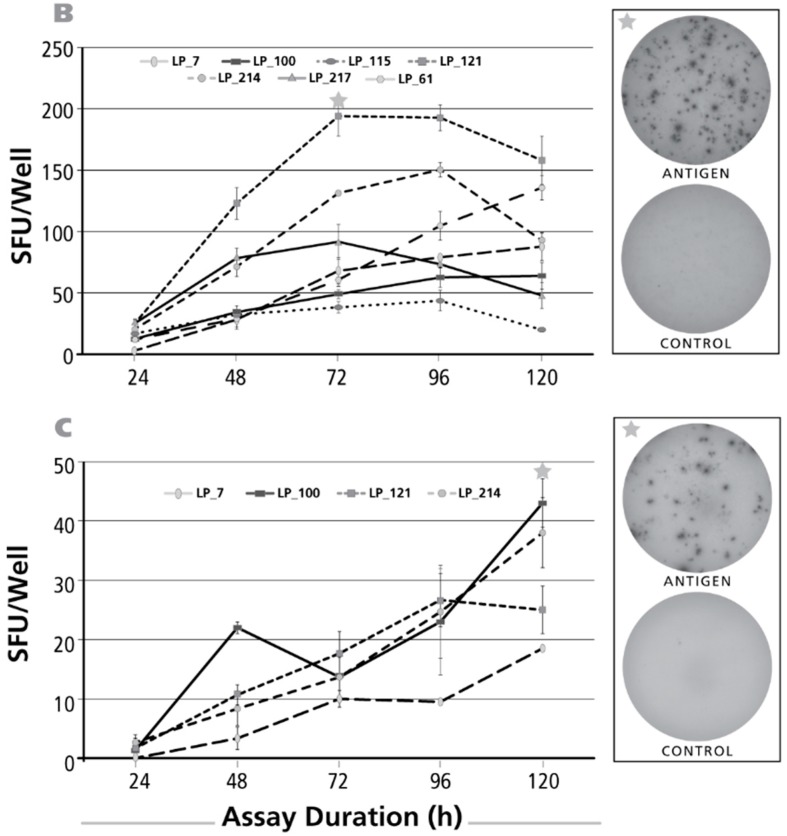
Kinetics of the antigen-induced IL-17 recall response. Donors and antigens were selected that scored IL-17 positive in the screening experiments (Figure 1). Panel (**A**) shows the test results for CMV gr.2 antigen-induced IL-17 production; in Panel (**B**) Purified Protein Derivative of PPD-triggered IL-17 was tested; in Panel (**C**) Dust mite was used as the recall antigen to activate IL-17-producing T cells. The donors tested are specified by symbols within each panel. The PBMC (400,000 cells per well) were cultured with the antigen for the time periods specified on the *X* axis (“Assay Duration”), and after the IL-17 spots were counted as SFU/well, *Y* axis. Each data point represents the mean and SD for the SFU counts in the three replicate wells tested. Of note, the time course was established testing the same cells in a single experiment: thus, for the different assay durations shown, only the length of the antigen stimulation period varied. On the right of each panel, on the top, the image of the respective antigen containing well is shown, with an asterisk marking the corresponding data point in the graph. The well image on the bottom shows the matching medium control well.

Of the antigens tested above, dust mite, PPD, and CMV gr.2 and were the ones to recall more frequently Th17 responses (Figure 1E). The kinetics of the IL-17 response induced by these antigens was tested for positive donors. The time course of the CMV gr.2 antigen-induced recall response is shown for four donors in Figure 2A. Borderline-to-no IL-17 production was seen at 24 h. The maximal numbers of SFU were elicited at 72–96 h, after which the number of SFU declined. Such decline in SFU is seen once the production of the analyte stops and it starts to dissociate from the membrane [32]. At peak SFU numbers, the IL-17 spots showed pristine morphology (an example of which is shown in the upper well image insert in Figure 2A). The corresponding medium control well is shown in the lower well insert of Figure 2A: in spite of the 96 h duration of the assay there was no background spot formation seen. The medium control wells contained no spots at 120 h, or the earlier time points, as well (data not shown). 

Results for the PPD-induced response are shown in Figure 2B. As with CMV gr.2 antigen, at the 24-h time point the numbers of SFU detected were a fraction of SFU present at later time points. While overall peak SFU formation was seen at 72 and 96 h as well, in two of seven donors SFU numbers continued to increase throughout the 120-h observation period. Representative PPD-induced IL-17 spots, and the corresponding medium control wells are shown as inserts in Figure 2B.

Also the dust mite antigen-induced IL-17 recall response was undetectable at 24 h for donors who scored positive at later time points. For three of the four donors, the numbers of SFU continued to rise up to 120 h, the longest incubation time tested. Only for one donor the peak was reached at 96 h. 

Overall, the above data shows that for all three recall antigens, and in all donors, a determination of antigen-specific Th17 cell numbers at 24 h, or earlier, completely underestimates the frequency of such cells, or fails to detect them altogether. Th17 cells can be accurately enumerated after a 72–96 h of activation period, occasionally even requiring 120 h stimulation, and more. All the above data have been generated using cryopreserved PBMC. As it seemed possible that the freeze-thawing process might impact the kinetics of cytokine production, we have systematically tested fresh and cryopreserved PBMC obtained from the same donors. No significant differences were found comparing the kinetics of recall antigen-induced IFN-γ, IL-2, IL-4, IL-5 and IL-17 production by fresh and cryopreserved PBMC (data not shown). 

### 3.3. Kinetics of the IL-5 Recall Response

**Figure 3:**
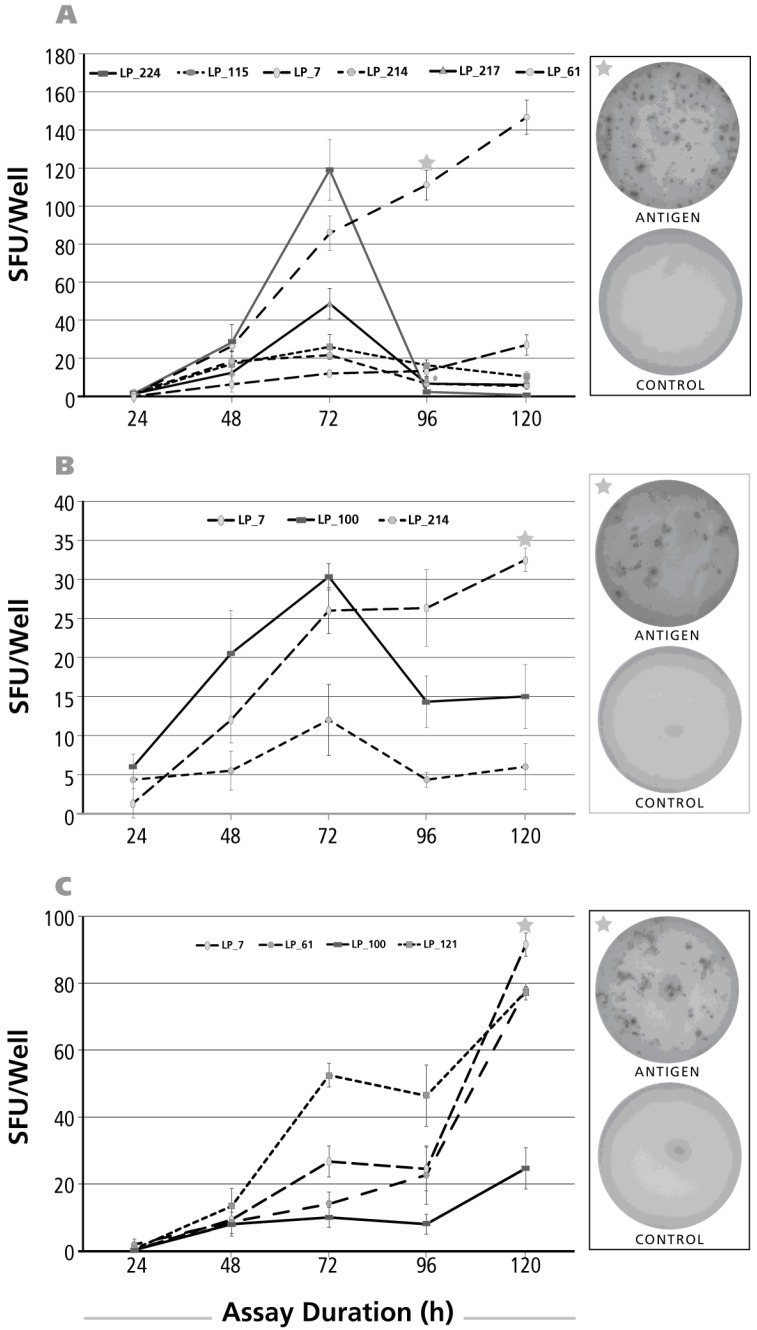
Kinetics of the IL-5 recall response by Th2 cells. The legend to Figure 2 applies, except that an IL-5 ELISPOT assay was done with donors and antigens that scored positive for IL-5. In Panel (**A**) CMV gr.2 was used as the recall antigen to activate IL-5 producing T cells; in Panel (**B**), PPD-triggered IL-5 production is shown; and in Panel (**C**) dust mite was used to stimulate T cells.

As for the IL-17 kinetics, PBMC donor/antigen combinations were selected that gave an IL-5 recall response. The results for CMV gr.2 antigen are shown in Figure 3A. No IL-5 SFU could be detected for an assay duration of 24 h. SFU numbers peaked after 72 h incubation, except for one of six donors in whom SFU numbers continued to rise up to the 120-h time point. The kinetics of the PPD-induced IL-5 response is shown in Figure 3B. Also for this antigen, peak SFU formation was seen after 72 h, with the exception of one donor in whom SFU numbers continued to rise until 120 h. Like for IL-17, the dust mite IL-5 recall response showed delayed kinetics with SFU numbers still rising at 120 h (Figure 3C).

In summary, for all three recall antigens, and in all donors tested, a 24-h determination of IL-5 fails to detect the presence of Th2 cells secreting this cytokine. While maximal SFU numbers were seen after 72 h of activation, occasionally even a 120-h stimulation period was required. Late determinations (96 and 120 h), while enhancing SFU numbers in some donors, can largely decrease in others (see Figure 3A), leading to their gross underestimation. For determining IL-5 SFUs, it therefore might be recommended to test both, the 72 and the 120 h time points. 

### 3.4. Kinetics of the IL-4 Recall Response

**Figure 4:**
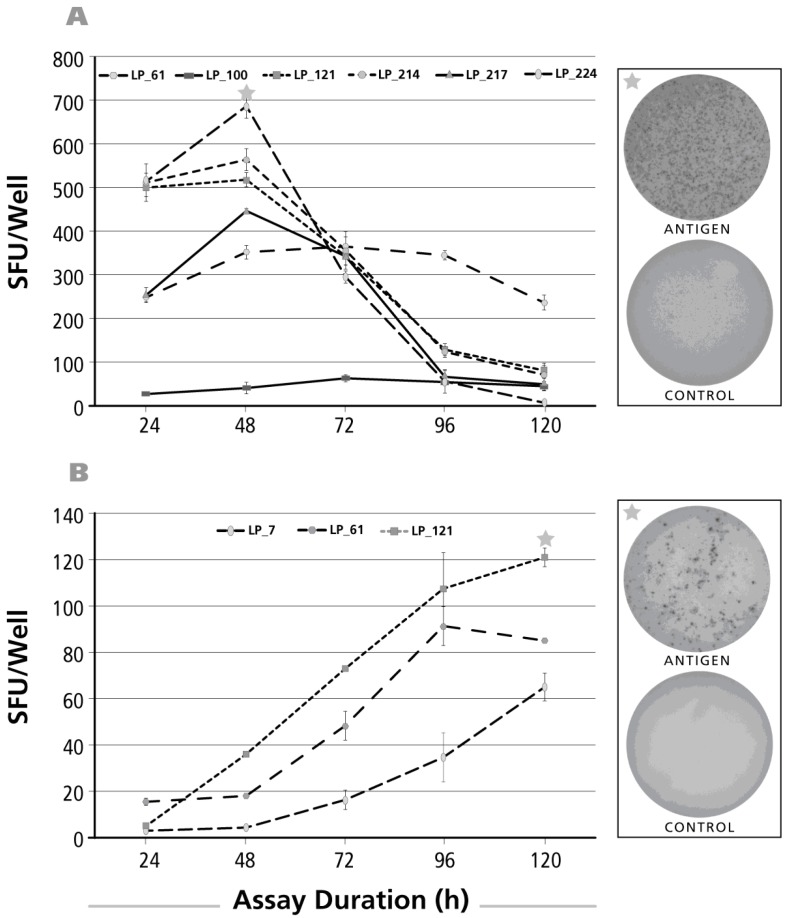
Kinetics of the IL-4 recall response by Th2 cells. The legend to Figure 2 applies, except that an IL-4 ELISPOT assay was done with donors and antigens that scored positive for IL-4. In Panel (**A**) CMV gr.2 was the recall antigen used to elicit IL-4 production in memory T cells; in Panel (**B**) the dust mite antigen stimulated IL-4 recall response is shown.

The kinetics of the IL-4 recall response was studied for CMV gr.2 (Figure 4A) and dust mite (Figure 4B) antigens. CMV gr.2-induced SFU formation was already detectable at the 24 h time point, but SFU numbers increased until 48 h, after which a rapid decline was seen for four of six donors. In two of six donors, SFU numbers reached a plateau by 96 h. Like with IL-17 and IL-5, also the dust mite antigen-induced IL-4 recall response showed a delayed kinetics (Figure 4B) with SFU numbers still continuing to rise up to the 120-h incubation period.

### 3.5. Kinetics of the IL-2 Recall Response

**Figure 5:**
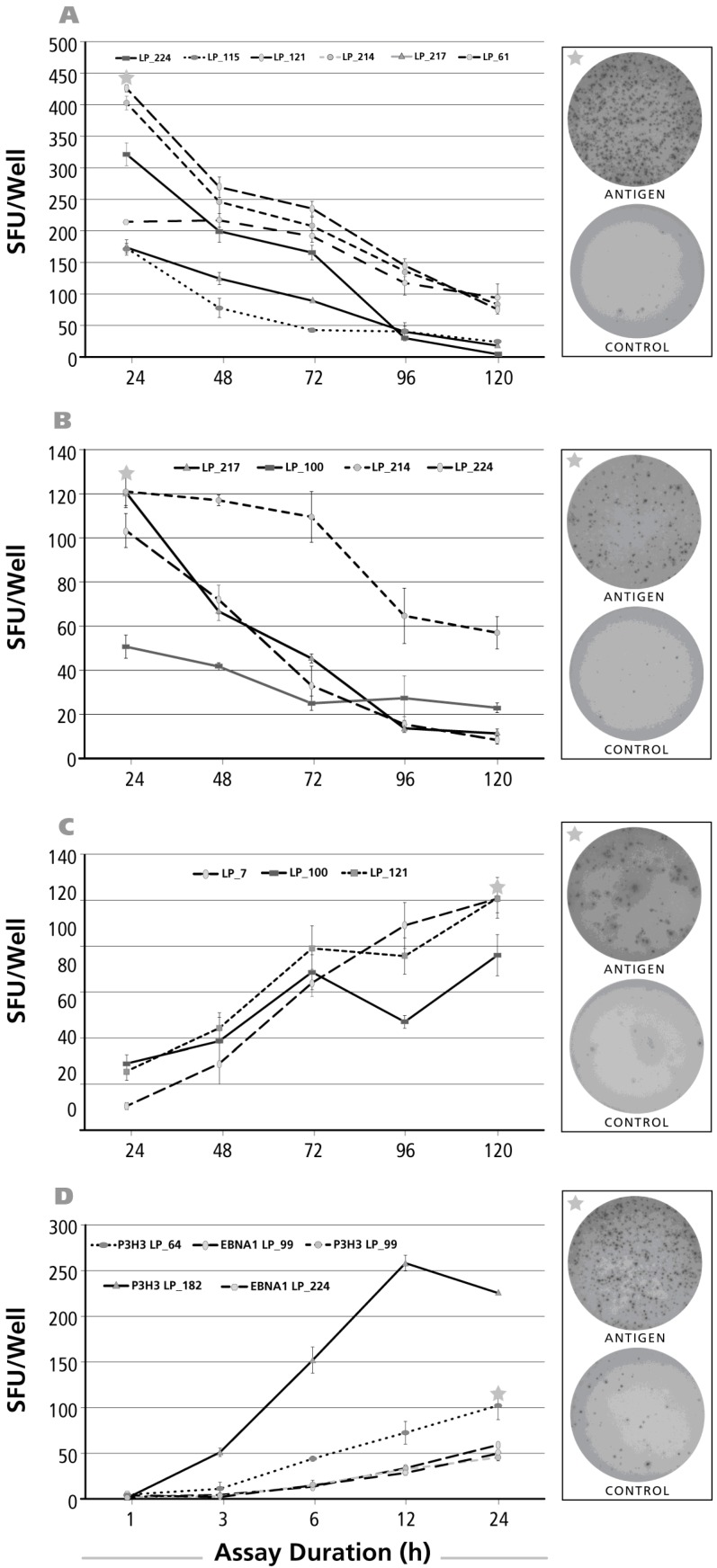
Kinetics of the IL-2 recall response by Th1 cells. The legend to Figure 2 applies, except that an IL-2 ELISPOT assay was done with donors and antigens that scored positive for IL-2. The antigens used to elicit IL-2 production were, in Panel (**A**); CMV gr.2; in Panel (**B**) PPD; and in Panel (**C**) dust mite; In Panel (**D**), the IL-2 assay duration was 1, 3, 6, 12 and 24 h with the insert specifying the donor/antigen combinations tested.

When the kinetics of the IL-2 recall response was studied in a similar fashion testing stimulation cultures of 24, 48, 72, 96 and 120 h duration, maximal SFU were detected already at the 24-h time point for CMV gr.2 (Figure 5A) and PPD (Figure 5B). Subsequently the numbers of SFU showed a rapid decline for both antigens. With the dust mite antigen, the opposite was observed: SFU numbers continued to rise up to 120 h (Figure 5C). 

The post-24 h decline of SFU numbers for CMV gr.2 and PPD suggested that IL-2 production might have peaked earlier. We therefore tested IL-2 SFU formation at earlier time points, 1, 3, 6, 12 and 24 h after the addition of antigen. Also, because of the highly discrepant kinetics seen above between dust mite vs. CMV gr.2 and PPD, we extended the study to test EBV antigens P3H3 and EBNA1. Increased numbers of IL-2 SFU became detectable 3–6 h after the addition of antigen, and steadily rose until the 24-h time point (Figure 5D). Jointly, these data suggest that the 24-h time point previously considered optimal for detecting T cell-mediated IL-2 production [33] holds up in our hands as well—while the delayed dust mite kinetics for IL-2, and for all the other cytokines tested, remains to be elucidated.

### 3.6. Kinetics of the IFN-γ Recall Response

**Figure 6:**
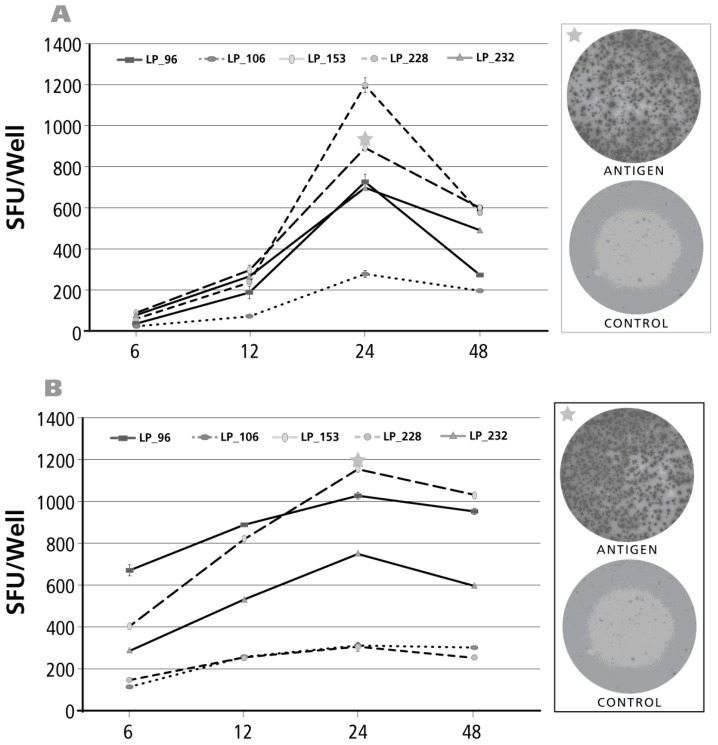

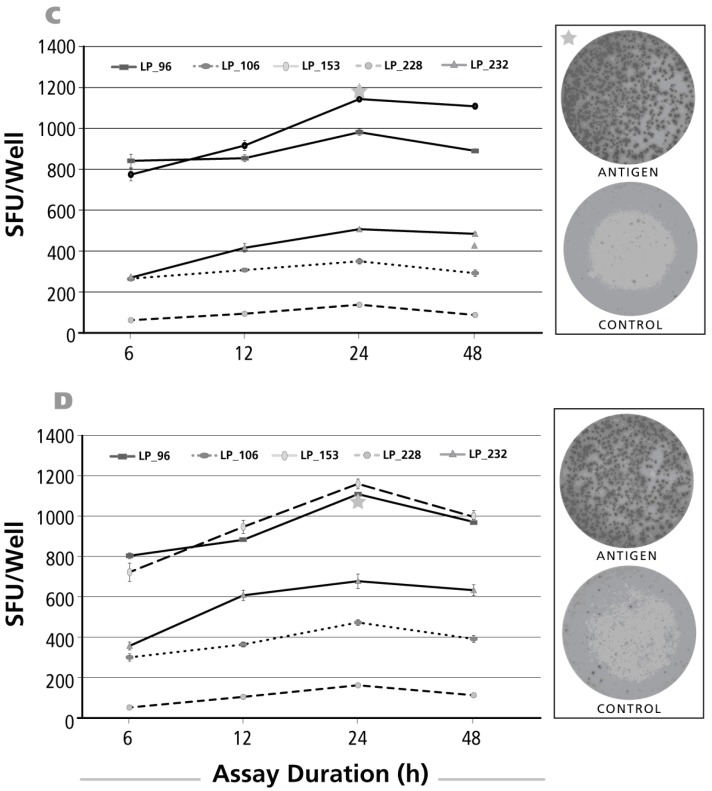
Kinetics of the IFN-γ recall response by Th1 cells. The legend to Figure 2 applies, except that an IFN-γ ELISPOT assay was performed with donors and antigens that scored positive for IFN-γ. Panel (**A**) shows CMV gr.2 antigen-triggered IFN-γ production that requires antigen processing; in Panel (**B**) the results for testing a pool of 15-mer peptides is shown—these peptides cover the sequence of CMV protein pp65—such peptides can bind directly to HLA-Class II molecules and do not need processing; Panel (**C**) shows the kinetics of IFN-γ production after stimulation with a nine amino acid long peptide of CMV that covers amino acid positions 495–503 of pp65 protein. This CMV pp65 (495–503) peptide is a well-defined HLA-A2-restricted CMV determinant and all donors tested in C were HLA-A2 positive; Panel (**D**) shows the kinetics of the CEF peptide pool induced IFN-γ recall response.

Unlike for the above cytokines, it is well established in the literature that antigen-induced IFN-γ production—when measured by ELISPOT—peaks at 24 h [19]. Measurements of Th1 cell frequencies by ICS, however, typically rely on 6-h antigen stimulation cultures [20]. This early time point is chosen due to the toxicity of secretion inhibitors. Do 6-h measurements underestimate the frequency of Th1 cells? To address this question, we studied the early kinetics of antigen-induced IFN-γ production. We compared CMV gr.2 antigen (Figure 6A), that needs to be processed before it can trigger CD4 cell activation, and a pool of 15-mer peptides that cover the CMV pp65 protein, HCMVA (pp65) (Figure 6B). The latter does not require further processing as the 15-mer peptides can bind directly bind to HLA Class II molecules and stimulate CD4 cells. Also a single HCMV peptide was studied, CMV pp65 (495–503), that represents a well-defined HLA-A2 restricted CMV determinant stimulating CD8 cells [28] (Figure 6C). We also included the CEF peptide pool in this study: it consists of 32 well-defined nine amino acid long peptide determinants of CMV, EBV and flu virus [29]. All peptide antigens elicited already close to maximal SFU numbers at 6 h relative to the 24-h time point although the latter represented the peak of IFN-γ production. In contrast, CMV gr.2 antigen-triggered IFN-γ production was still barely detectable at 6 h, by far suboptimal even at 12 h, and peaked after an assay duration of 24 h. Therefore, for immune monitoring purposes, IFN-γ measurements 6 h after peptide stimulation night be suitable, but are still suboptimal for measuring Th1 cell frequencies. When additional antigen processing is required, due to the additional time needed for antigen processing and presentation to occur, 6-h measurements can completely fail to detect the antigen-specific Th1 cells. In the latter case, like also for peptide-triggered test systems, the 24-h time point seems to be optimal to accurately enumerate frequencies of antigen-specific IFN-γ producing T cells within PBMC. 

## 4. Conclusions

Our data shows that Th1, Th2 and Th17 cells do not produce cytokine synchronously. The secretion of IFN-γ by Th1 cells peaks 24 h after antigen encounter. While peptide-triggered IFN-γ production by these cells can reach close to maximal levels at 6 h—the time point typically used for detecting this cytokine by ICS, it requires 24 h for protein antigen that—unlike peptides—needs to be internalized, processed and transported on HLA Class II molecules to the surface of the APC before the antigen becomes available for T cell recognition. IL-2 production by Th1 cells also peaks at 24 h. For Th2 cells, the kinetics of IL-4 and IL-5 production showed marked differences. IL-4 production was detectable already at 24 h, but peaked at 48 h, whereas IL-5 secretion was barely detectable even at 48 h, and peaked at 72 h, and in some instances even later. The IL-17 secretion kinetics by Th17 cells was similar to the one of IL-5. A striking observation was that these kinetics can have antigen-dependent variations. The likely explanation for this finding is that—unlike peptides—complex antigens acquired by APC from the extracellular space are not internalized, processed and presented at the same rate. The APCs’ antigen uptake by pinocytosis will be substantially less efficient and slower than internalization of an antigen that binds to receptors on these cells. The pathway of antigen uptake can also impact the lysosomal processing of antigen, whereby receptor-mediated uptake is likely to activate the APC and the processing machinery, whereas pinocytotic uptake will leave the APC in a resting state. APC activated by antigen will also upregulate the expression of their cell adhesion and costimulatory molecules, thereby enhancing antigen presentation—unactivated APC will not do so. Some complex antigens, as used here, might even actively inhibit antigen processing/presentation. This seems to be the case for dust mite antigen, which causes delayed T cell responses for all cytokines tested. 

Considerable interindividual variations have been seen in the kinetics of the T cell recall responses, even to the same antigen. These are likely to result from allelic polymorphisms affecting key molecules involved in the antigen processing and presentation machinery, not T cell activation itself. The frequency of the relevant alleles in the test population would then define the numbers of donors that need to be tested to establish the variation in kinetics. 

Overall, the data highlights the necessity of establishing for each complex antigen the kinetics of cytokine production byT cells before testing a larger cohort of human donors for the recall response to that antigen.
